# Spatial patterning and correlates of self-harm in Manchester, England

**DOI:** 10.1017/S2045796019000696

**Published:** 2019-11-19

**Authors:** Chien-Yu Lin, Harriet Bickley, Caroline Clements, Roger T. Webb, David Gunnell, Chia-Yueh Hsu, Shu-Sen Chang, Nav Kapur

**Affiliations:** 1 Institute of Health Behaviors and Community Sciences, College of Public Health, National Taiwan University, Taipei, Taiwan; 2 Graduate School of Sport Sciences, Waseda University, Tokorozawa, Japan; 3 Division of Psychology & Mental Health, Centre for Mental Health and Safety, The University of Manchester, Manchester, UK; 4 Manchester Academic Health Sciences Centre (MAHSC), Manchester, UK; 5 National Institute for Health Research Greater Manchester Patient Safety Translational Research Centre, Manchester, UK; 6 Bristol Medical School, Population Health Sciences, University of Bristol, Bristol, UK; 7 National Institute for Health Research Biomedical Research Centre at the University Hospitals Bristol NHS Foundation Trust and the University of Bristol, Bristol, UK; 8 Department of Psychiatry, Wan Fang Hospital, Taipei Medical University, Taipei, Taiwan; 9 Greater Manchester Mental Health NHS Foundation Trust, Manchester, UK; 10 Department of Psychiatry, School of Medicine, College of Medicine, Taipei Medical University, Taipei, Taiwan; 11 Psychiatric Research Center, Wan Fang Hospital, Taipei Medical University, Taipei, Taiwan

**Keywords:** Deprivation, self-harm, social fragmentation, socioeconomic characteristics, spatial analysis

## Abstract

**Aims:**

To investigate the spatial distribution of self-harm incidence rates, their socioeconomic correlates and sex/age differences using data on self-harm presentations to emergency departments from The Manchester Self-Harm Project (2003–2013).

**Methods:**

Smoothed standardised incidence ratios for index self-harm episodes (*n* = 14 771) and their associations with area-level socioeconomic factors across 258 small areas (median population size = 1470) in the City of Manchester municipality were estimated using Bayesian hierarchical models.

**Results:**

Higher numbers and rates of self-harm were found in the north, east and far southern zones of the city, in contrast to below average rates in the city centre and the inner city zone to the south of the centre. Males and females aged 10–24, 25–44 and 45–64 years showed similar geographical patterning of self-harm. In contrast, there was no clear pattern in the group aged 65 years and older. Fully adjusted analyses showed a positive association of self-harm rates with the percentage of the unemployed population, households privately renting, population with limiting long-term illness and lone-parent households, and a negative association with the percentage of ethnicity other than White British and travel distance to the nearest hospital emergency department. The area-level characteristics investigated explained a large proportion (four-fifths) of the variability in area self-harm rates. Most associations were restricted to those aged under 65 years and some associations (e.g. with unemployment) were present only in the youngest age group.

**Conclusions:**

The findings have implications for allocating prevention and intervention resources targeted at high-risk groups in high incidence areas. Targets for area-based interventions might include tackling the causes and consequences of joblessness, better treatment of long-term illness and consideration of the accessibility of health services.

## Introduction

At least 800 000 people die by suicide worldwide every year; approximately 6000 of these deaths occur in the UK. Amongst people presenting to hospitals with self-harm, the risk of dying by suicide is approximately 50 times that in the general population (Hawton *et al*., 2015). There are more than 200 000 self-harm presentations to hospitals each year in England (Hawton *et al*., 2007) and an estimated cost of £162 million per year due to the hospital management of self-harm (Tsiachristas *et al*., 2017). Furthermore, self-harm causes significant distress and costs not only to the people who have self-harmed but also their friends and families (Ferrey *et al*., 2016). Self-harm hospital presentations therefore represent both an opportunity for suicide prevention and an important target for intervention in its own right.

An important consideration for the prevention and intervention of self-harm is the identification of areas with elevated self-harm rates. Such investigations contribute to a better understanding of factors that may influence geographical variations in self-harm rates as well as inform resource allocation. However, previous research into the geographical distribution of self-harm is limited and yields inconsistent findings. For example, in London, Canada, rates of self-harm were highest in the city centre and decreased as the distance from the centre increased (Jarvis *et al*., 1982); by contrast, in a recent study from London, England, self-harm rates were lower in areas closer to the city centre (Polling *et al*., 2019). Furthermore, another England-wide small-area analysis showed a non-linear association between a rurality indicator and rates of hospitalised self-harm episodes, with the highest rates occurring in suburban areas with intermediate rurality scores (Congdon, 2013). These studies also revealed different socioeconomic factors that were associated with area self-harm rates, such as living arrangements (Jarvis *et al*., 1982) or deprivation (Congdon, 2013; Polling *et al*., 2019). Two recent systematic reviews indicated that area socioeconomic deprivation is positively associated with the rate of suicidal behaviour (Burrows and Laflamme, 2010; Cairns *et al*., 2017). Other area-level characteristics such as social fragmentation (Congdon, 1996; O'Farrell *et al*., 2015), ethnic minority (Neeleman *et al*., 2001) and travel time to the nearest emergency departments (O'Farrell *et al*., 2015) were also found to be associated with self-harm rates.

A few previous studies suggested that the associations between self-harm rates and area-level characteristics varied by sex and age. A study from Oxfordshire, England, showed that socioeconomic deprivation was associated with increased rates of self-harm in both males and females, whilst social fragmentation was only associated with increased female self-harm rates (Harriss and Hawton, 2011). Studies from Canada and Ireland showed a steeper gradient for self-harm rates from the least to most deprived areas amongst the younger group than the elderly (Burrows *et al*., 2010; O'Farrell *et al*., 2015). However, to the best of our knowledge, there have been no previous investigations into sex- and age-specific patterns of both spatial distributions and correlates of self-harm.

The City of Manchester municipality (population = 503 000 in 2011) in northern England represents a unique setting to study geographical variations in self-harm. A substantial proportion of the city's population are in the most deprived localities in England, but there are also large variations in area deprivation within the city. In 2007, 52% of its 259 Lower Super Output Areas (LSOAs, a small area unit of a geographical hierarchy based on the aggregates of postcodes, defined by the UK Office for National Statistics) were amongst the most deprived 10% in England; in contrast, 5% were classified as the least deprived 50% (Manchester City Council, 2008). The population comprises a highly diverse mix of ethnic groups (41% were of an ethnicity other than White British in 2011) (ESRC Centre on Dynamics of Ethnicity (CoDE), 2013). The only previous analysis of area-level characteristics and self-harm in the City of Manchester population was based on the data from a period nearly two decades ago (1997–2002), conducted at a relatively large geographical unit (i.e. census area statistics ward [casward]; *n* = 33) (Johnston *et al*., 2006). The study showed a high correlation between area deprivation and self-harm but did not take into consideration potential confounders, the spatial patterning of self-harm or any sex/age differences.

In the present study, we investigated the spatial patterning of overall, sex- and age-specific, and method-specific rates of self-harm across small areas (*n* = 258) in the City of Manchester population during 2003–2013. We also examined the associations between a wide range of area-level characteristics and overall and sex- and age-specific self-harm rates. We used data from the Manchester Self-Harm Project, which comprehensively collects data for all self-harm presentations to emergency departments (rather than just self-harm hospitalisations as in some previous studies, e.g. Congdon, 2013) in the city that allows a systematic investigation into small-area variations in self-harm.

## Materials and methods

### Self-harm episode data

Data for self-harm were extracted from the Manchester Self-Harm Project for individuals presenting to hospitals providing emergency care in the City of Manchester, North West of England, during 2003–2013. The Project was established in 1997 to monitor hospital presentations following self-harm (Cooper *et al*., 2005; Bickley *et al*., 2013). Information on sex, age and the timing and method of self-harm was collected for all self-harm presentations via emergency department patient records. Self-harm was defined as intentional self-poisoning or self-injury, irrespective of the motivation and degree of suicidal intent (Hawton *et al*., 2003). A previous local audit of patient ‘flows’ across municipal boundaries estimated that presentations for self-harm at the three hospitals accounted for more than 90% of all those made by residents of the municipality (Kapur *et al*., 2013). We analysed data for the first self-harm episode for each person during the study period (the index episode), as multiple self-harm episodes by the same individual may generate an overestimated incidence rate in the area where that person lived. Each index self-harm episode was assigned to one of the LSOAs (*n* = 258) according to the postcode of residence. The number of LSOAs in the City of Manchester municipality increased from 259 in 2001 to 282 in 2011; we created 258 LSOAs with consistent boundaries over the study period. Individuals with no fixed abode (*n* = 366) or with a postcode outside the City of Manchester (*n* = 7086) municipal boundary were excluded.

We used mid-2008 (i.e. the midpoint of the study period) population estimates for LSOAs in the City of Manchester municipality as the population denominator; data were from the Office for National Statistics (Office for National Statistics, 2019). The median population aged 10 years and above for LSOAs was 1470 (interquartile range: 297; Q1–Q3 = 1362–1659).

### Data for area-level characteristics

The majority of area-level socioeconomic characteristics were extracted from the 2001 national census. Data for the Index of Multiple Deprivation (IMD), as used in the previous studies (Johnston *et al*., 2006), were not used in the main analysis of the present study as the index includes component variables (e.g. emergency admissions to hospital) that would reflect the local rates of self-harm hospital presentations, and it is inappropriate to include exposure variables that contain information of the outcome variable of interest in the analysis. However, we conducted sensitivity analyses using the IMD 2010 (https://www.gov.uk/government/statistics/english-indices-of-deprivation-2010) to examine the robustness of the findings. Travel distance from the centroid of LSOAs to the nearest emergency department by driving based on data from Google Maps was extracted from a website (https://www.doogal.co.uk/drivingdistances.php).

#### Socioeconomic deprivation

The Townsend deprivation index is a widely used composite deprivation measure derived from four census variables (Townsend *et al*., 1988): the percentage of (i) households without a car; (ii) households not owner-occupied (households where the occupants did not own their home); (iii) unemployed population; and (iv) overcrowded households (i.e. households with more than one person per room). The standardised scores (*z*-scores) of these four variables were summed to obtain a single value which is the Townsend deprivation index, indicating the relative level of material deprivation within a population.

#### Social fragmentation

Congdon's social fragmentation (‘anomie’) score, which was developed based on Durkheim's theory of social integration, is a composite measure that includes the following four census variables (Congdon, 2004): the percentages of (i) population whose residences changed within 1 year (an indicator of population mobility); (ii) single-person households; (iii) households privately renting; and (iv) unmarried adults (i.e. single and divorced/widowed). The social fragmentation score for each LSOA was calculated by summing the standardised scores of the four area-level characteristics.

#### Other area-level characteristics

The following variables were included in the analysis based on previous studies that reported on their ecological associations with area rates of self-harm or suicide: the social class distribution of households (Middleton *et al*., 2004), social housing (i.e. low-cost rental housing owned or managed by the state or non-profit organisations) (Hsu *et al*., 2015), population with limiting long-term illness (Middleton *et al*., 2004), lone-parent households (Middleton *et al*., 2004), ethnicity other than White British (Johnston *et al*., 2006), population density (O'Farrell *et al*., 2015) and travel distance to the nearest emergency department (O'Farrell *et al*., 2015).

### Statistical analyses

Sex- and age-standardised incidence ratios (SIRs), using 5-year age bands, for index self-harm episodes were calculated amongst people aged 10 and above for each LSOA in the City of Manchester municipality during 2003–2013. We also calculated SIRs for males and females aged 10–24, 25–44, 45–64 and 65 years and over, respectively. We considered the primary method of self-harm based on the likelihood of fatality (e.g. self-poisoning takes precedence over self-cutting) and calculated SIRs for self-harm by method (self-poisoning, self-cutting and others such as hanging, traffic-related self-harm, hitting something and head banging). A SIR of one represents an area with a rate of self-harm that is the same as that across the City of Manchester as a whole; an area with a SIR <1 suggests that the area has lower than expected incidence of self-harm and the opposite is true for areas with a SIR >1. The ratio between smoothed SIR values at 95 and 5% (‘mid-90% ratio’) was calculated, with a higher value indicating a higher level of geographical variation.

Data over the 11-year study period were aggregated to ensure a statistically sufficient number of self-harm events in small areas. However, the low number of self-harm episodes in small areas, particularly in sex- and age-specific groups, may still lead to statistical instability in the estimates of SIRs. We thus used Bayesian hierarchical models to estimate the ‘smoothed’ SIRs for each LSOA and the associations of area-level characteristics with self-harm rates. The Bayesian hierarchical model is based on a Poisson assumption for the observed number of self-harm episodes with two random effects accounting for the heterogeneity across areas in the whole study region (unstructured variability) and the heterogeneity amongst the neighbouring areas (structured variability) (Besag *et al*., 1991; Congdon, 1997). Non-informative prior distributions were used in the estimation of the Bayesian models; for example, the standard errors of unstructured and structured variability were specified using a uniform distribution (0, 5) in the analysis (Gelman, 2006). Neighbouring areas were defined as those sharing a common boundary.

We used standardised values of raw area-level characteristics, or their log-transformed values if the raw values were skewed, in the analyses. Spearman's correlation was used to examine the correlations amongst area-level characteristics (online Supplementary Appendix Table 1). Variance inflation factors (VIFs) were calculated, with a value above 10 indicating a high level of multicollinearity (online Supplementary Appendix Table 2) (James *et al*., 2013). In sensitivity analyses, we omitted variables from the regression models that were of high multicollinearity. We examined the linearity of associations by comparing the models that included area-level characteristics as a categorical variable (i.e. the quartiles) and as a continuous variable (i.e. the *z* scores) based on the deviance information criterion (DIC) (Spiegelhalter *et al*., 2002), with a lower DIC value indicating a better model fit. The DIC values were also calculated and compared between the two models, i.e. one with the deprivation and social fragmentation indices but not their component variables, and the one with the component variables but not the indices. Bayesian hierarchical models were estimated using the Markov-Chain Monte Carlo method (Gilks *et al*., 1996) in WinBUGS version 1.4 (Spiegelhalter *et al*., 2003). Visual inspection of three chains and the Gelman–Rubin diagnostic (Gelman, 2006) were used to examine the convergence of models; values of the *R*-statistic >1.2 indicate poor convergence. To investigate the spatial autocorrelation/clustering of self-harm, we calculated Moran's *I* statistics using a method that considers the different population sizes across areas in GeoDa (Anselin *et al*., 2006). A value of zero indicates no spatial autocorrelation, while positive and negative values indicate positive or negative spatial autocorrelations, respectively (Moran's *I* could range from −1 to 1).

### Mapping

**Fig. 1.**
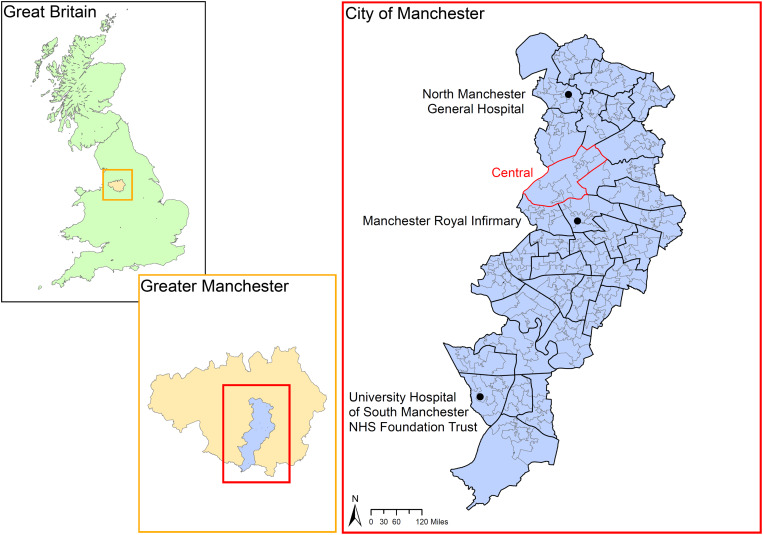
Location of the study region. *Note*: Boundaries were based on the 2001 Census. The boundaries of census area statistics ward (casward; *n* = 33) were highlighted in bold black. The boundaries of the ‘Central’ casward (i.e. the city centre) were highlighted in bold red. The locations of the three study hospitals were marked. Source of digitised boundary data: UK Data Service (https://borders.ukdataservice.ac.uk/index.html).

Figure 1 shows the geographical location of the study region, namely the City of Manchester, a municipality that stretches from the middle to the southern border of the conurbation of Greater Manchester, England. The municipality boundary included the ‘city centre’, indicated as the ‘Central’ casward in Fig. 1, and, relative to the city centre, areas referred to as the north, east, south and far southern zones of the city.

The spatial distribution of SIRs for self-harm was presented using choropleth maps with seven category breaks and a divergent red-blue colour scheme (Brewer, 1996). A cartogram was produced to highlight the geographical distribution of the burden of self-harm; the size of LSOAs was rescaled proportionally to the number of index self-harm episodes on the map (Gastner and Newman, 2004). All maps were created using ArcGIS Version 10.5.

## Results

In 2003–2013, there were 14 771 index self-harm episodes amongst people aged 10 years and above (males 42.6%) in the City of Manchester municipality. Amongst males, those aged 10–24, 25–44, 45–64 and 65 years and older accounted for 33.3, 48.3, 15.9 and 2.4% of all index self-harm episodes, respectively; the corresponding figures for females were 46.0, 38.5, 13.7 and 1.8%, respectively. Self-poisoning accounted for most (79.2%) self-harm episodes, followed by self-cutting (14.1%) and other methods (6.7%).

### Spatial patterning of index self-harm episodes

Unsmoothed SIRs for self-harm showed marked variations across the 258 small areas even after excluding the 10% extreme values (a nearly fivefold difference in the mid-90% ratio; range 0.38–1.87). Smoothed SIRs for self-harm also showed a fourfold difference in the mid-90% ratio (range 0.45–1.84) (online Supplementary Appendix Table 3). Moran's *I* of the self-harm SIRs was 0.51 (*p* < 0.001), indicating moderate spatial autocorrelation of self-harm rates between neighbouring areas.

**Fig. 2.**
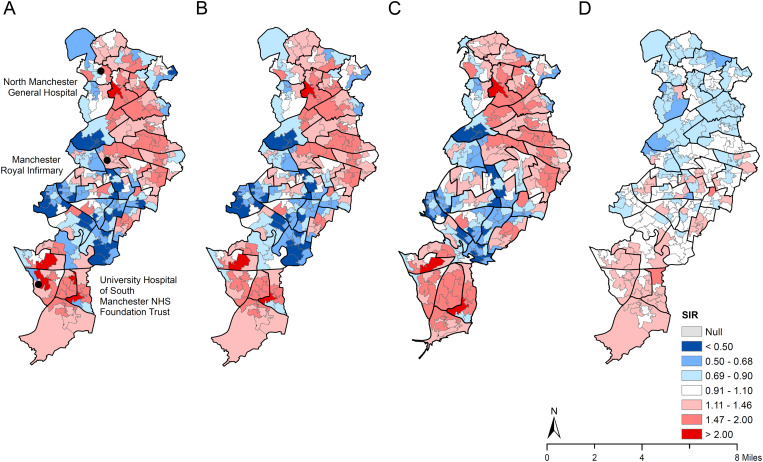
Maps of standardised incidence ratios (SIRs) for index self-harm episode in people aged 10 years or above across 258 Lower Super Output Areas (LSOAs) in the City of Manchester municipality, 2003–2013: (*a*) raw SIRs; (*b*) smoothed SIRs estimated using Bayesian hierarchical models; (*c*) a cartogram of smoothed SIRs with the LSOA size rescaled in proportion to the number of index self-harm episodes in each LSOA; and (*d*) residual SIRs after adjusting for 15 area socioeconomic characteristics. *Note*: The boundaries of census area statistics ward (casward; *n* = 33) were highlighted in bold black.

Figure 2 shows the maps of self-harm SIRs. The spatial patterning of unsmoothed SIRs (Fig. 2*a*) and smoothed SIRs (Fig. 2*b*) was very similar. There were above average self-harm rates in the north, east and far southern zones of the municipality. By contrast, the city centre and inner city zone to the south of the centre showed generally below average self-harm rates. The cartogram showed the concentration of self-harm burden in the same high self-harm rate areas (Fig. 2*c*). Based on the posterior estimates of smoothed SIRs, we calculated the posterior probability of SIRs >1 (i.e. the probability of above-average rates, ranging from 0 to 1) for each LSOA and produced a map (online Supplementary Appendix Fig. 1); the map shows high probability (>0.8) in the same regions with above-average rates of self-harm as shown in Fig. 2*b*.

**Fig. 3.**
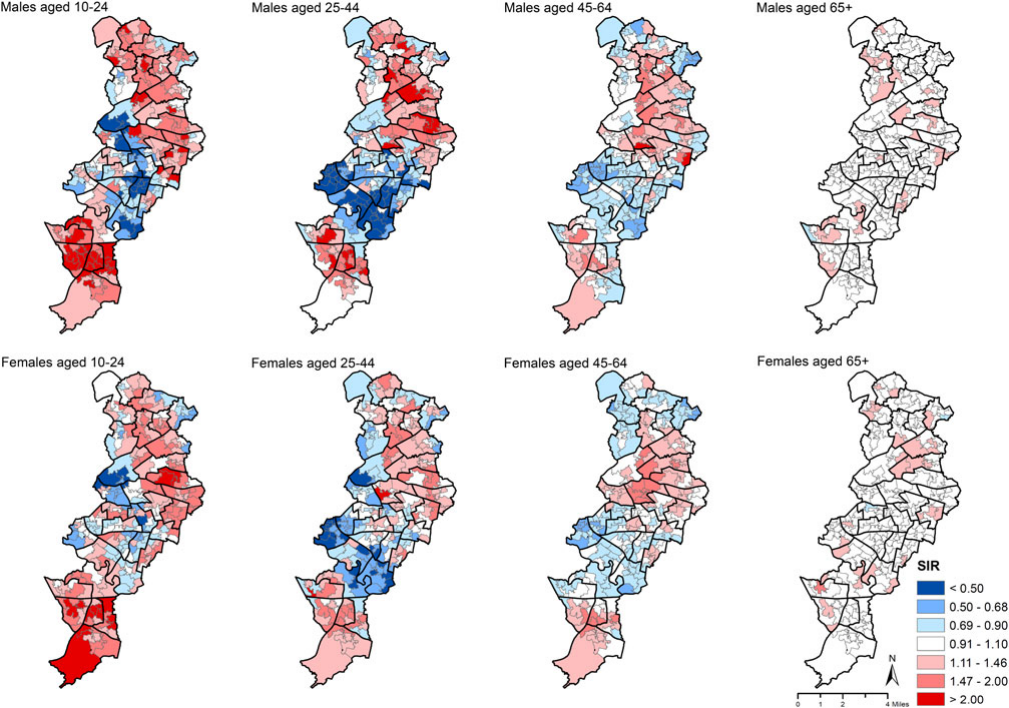
Maps of smoothed standardised incidence ratios (SIRs) for index self-harm episode in males and females aged 10–24, 25–44, 45–64 and 65+ years across 258 Lower Super Output Areas (LSOAs) in the City of Manchester municipality, 2003–2013. *Note*: The boundaries of census area statistics ward (casward; *n* = 33) were highlighted in bold black.

Overall, males showed larger geographical variations in self-harm rates (a 5.30-fold difference in the mid-90% ratio) than females (a 3.81-fold difference), with the greatest variations observed in males aged 25–44 (6.61-fold). There was spatial autocorrelation in both males (Moran's *I* = 0.48, *p* < 0.001) and females (Moran's *I* = 0.40, *p* < 0.001); males aged 25–44 years showed a higher level of spatial autocorrelation (Moran's *I* = 0.43, *p* < 0.001) than other sex/age groups (Moran's *I* ranged from −0.01 to 0.30). By contrast, there was weak or no statistical evidence for spatial autocorrelation in elderly males and females (online Supplementary Appendix Table 3). Generally, sex- and age-specific groups showed similar spatial patterning to the overall spatial patterning, with the only exception being elderly people showing no clear geographical patterns of self-harm rates (Fig. 3). The spatial patterning of self-harm was similar across different self-harm methods (online Supplementary Appendix Fig. 2).

### Spatial correlates of index self-harm episodes

The spatial distributions of area-level characteristics studied are shown in online Supplementary Appendix Fig. 3. Table 1 shows the associations of self-harm rates with area-level characteristics. In the unadjusted models, all explanatory variables investigated were associated with self-harm rates, with the two exceptions of the social fragmentation composite score and travel distance to the nearest emergency department. Socioeconomic deprivation (the Townsend deprivation index and all of its four component variables) was positively associated with self-harm rates; by contrast, there were some inconsistent associations between social fragmentation component variables and self-harm rates (i.e. a negative association of self-harm with population mobility and households privately renting *v*. a positive association with single-person households and unmarried adults).

**Table 1.** Rate ratios (RR) and 95% credible intervals (CrI)^a^ of index self-harm incidence (in people aged 10 or more years) associated with one standard deviation increase in levels of each of the area socioeconomic characteristics across 258 Lower Super Output Areas in the City of Manchester municipality, 2003–2013

	Unadjusted		Model 1 Adjusted for 9 variables		Model 2 Adjusted for 15 variables	
	RR	95% CrI	RR	95% CrI	RR	95% CrI
Townsend deprivation index	**1.09**	**(1.08, 1.11)**	**1.06**	**(1.02, 1.11)**		
Households without a car (%)	**1.39**	**(1.32, 1.46)**			1.01	(0.88, 1.15)
Households not owner-occupied (%)	**1.33**	**(1.27, 1.39)**			0.93	(0.78, 1.13)
Unemployed population (%)	**1.34**	**(1.28, 1.41)**			**1.10**	**(1.02, 1.19)**
Overcrowded households (%)^b^	**1.17**	**(1.10, 1.24)**			1.03	(0.96, 1.12)
Social fragmentation score	1.01	(0.99, 1.03)	0.99	(0.96, 1.02)		
Population mobility (%)^b^	**0.91**	**(0.85, 0.98)**			0.99	(0.91, 1.07)
Single-person households (%)^b^	**1.16**	**(1.10, 1.21)**			1.06	(0.99, 1.14)
Households privately renting (%)^b^	**0.86**	**(0.81, 0.93)**			**1.20**	**(1.08, 1.32)**
Unmarried adults (%)^b^	**1.17**	**(1.10, 1.24)**			0.95	(0.86, 1.05)
Social class IV and V households (%)^c^	**1.38**	**(1.31, 1.45)**	0.92	(0.84, 1.02)	0.98	(0.88, 1.10)
Social housing (%)	**1.34**	**(1.29, 1.40)**	0.94	(0.86, 1.02)	1.18	(0.99, 1.42)
Population with limiting long-term illness (%)	**1.38**	**(1.32, 1.45)**	**1.25**	**(1.17, 1.32)**	**1.17**	**(1.09, 1.25)**
Lone-parent households (%)	**1.31**	**(1.25, 1.37)**	**1.19**	**(1.11, 1.27)**	**1.17**	**(1.09, 1.26)**
Population other than White British (%)	**0.87**	**(0.79, 0.95)**	**0.88**	**(0.83, 0.93)**	**0.84**	**(0.79, 0.90)**
Population density (per hectare)	**0.95**	**(0.90, 1.00)**	1.00	(0.96, 1.03)	1.00	(0.96, 1.04)
Travel distance to the nearest emergency department by driving	0.95	(0.86, 1.06)	**0.91**	**(0.86, 0.95)**	**0.91**	**(0.86, 0.95)**

a The 95% credible intervals of rate ratios that do not include one are highlighted in bold.

b These variables were firstly log-transformed because of their skewed distributions.

c Based on the occupational status of household reference person; IV: semi-skilled and unskilled manual occupations; V: on state benefit, unemployed and lowest grade occupations.

In the adjusted models that included Townsend deprivation index and social fragmentation score, but not their component variables, and other area-level characteristics, self-harm rates were positively associated with Townsend deprivation index but not associated with the social fragmentation score (Model 1 in Table 1). In the fully adjusted model that included component variables of the Townsend deprivation index and social fragmentation score, the associations found in unadjusted models were mostly attenuated (Model 2 in Table 1). After adjusting for all other variables, there were positive associations with one component deprivation variable (the percentage of unemployed population; RR = 1.10, 95% CrI 1.02–1.19), one component social fragmentation variable (households privately renting: RR = 1.20, 95% CrI 1.08–1.32), population with limiting long-term illness (RR = 1.17, 95% CrI 1.09–1.25) and lone-parent households (RR = 1.17, 95% CrI 1.09–1.26), and negative associations with ethnicity other than White British (RR = 0.84, 95% CrI 0.79–0.90) and travel distance to the nearest emergency department by driving (RR = 0.91, 95% CrI 0.86–0.95). Model 2 (DIC = 1908.1) showed a better fit than Model 1 (DIC = 1912.9), indicating a better performance for the model with the component variables of Townsend deprivation index and social fragmentation score than that with the aggregate scores. Sensitivity analyses using the IMD 2010 as the deprivation index showed similar findings – the IMD and two of its domain variables (‘employment deprivation’ and ‘health deprivation and disability’) were associated with LSOA self-harm rates in adjusted analyses (online Supplementary Appendix Table 4).

There was no evidence that the results of multivariable regression analyses were affected by multicollinearity between area-level variables. In sensitivity analyses that stepwise omitted variables showing the highest multicollinearity levels until all variables had a VIF below 10, the results of the final model were similar (online Supplementary Appendix Table 2). There was no evidence for non-linear associations between the area-level variables examined and self-harm rates, except the percentage of the unemployed population and social class IV and V households (online Supplementary Appendix Table 5).

The area-level characteristics investigated explained a high percentage (80.6%) of the variability in area self-harm rates, based on comparing the estimates of geographical variability in the constant-only models and the fully adjusted models. Figure 2*d* presents the map of residual SIRs for self-harm after accounting for all studied variables. In the residual map, the concentration of areas with high self-harm rates was mostly attenuated, particularly in the north and east zones of the municipality, whilst there was still some concentration of high rates in the districts furthest south, indicating that some other area-level factors not examined may underlie increased self-harm rates in these areas.

Table 2 shows fully adjusted sex- and age-specific results of the regression analyses. Overall, the patterns were similar in males and females but there were differences between age groups. The associations of the percentage of the unemployed population and population with limiting long-term illness with self-harm rate were found only in the youngest groups aged 10–24 years. The association with households privately renting was found in males aged 45–64 and females aged 10–24. The associations with lone-parent households, ethnicity other than White British and travel distance to the nearest emergency department (particularly in males) were mainly observed in younger groups.

**Table 2.** Rate ratios (RR) and 95% credible intervals (CrI)^a^ of index self-harm episode in males and females aged 10–44, 45–64 and 65+ years associated with one standard deviation increase in levels of each of the area socioeconomic characteristics after controlling for all other variable across 258 Lower Super Output Areas in the City of Manchester municipality, 2003–2013

	Male								Female							
	Aged 10–24		Aged 25–44		Aged 45–64		Aged 65 +		Aged 10–24		Aged 25–44		Aged 45–64		Aged 65 +	
Townsend deprivation index																
Households without a car (%)	1.06	(0.78, 1.38)	1.20	(0.96, 1.47)	1.06	(0.76, 1.46)	1.12	(0.49, 2.21)	1.02	(0.80, 1.30)	0.90	(0.72, 1.11)	1.01	(0.75, 1.35)	1.59	(0.70, 3.11)
Households not owner-occupied (%)	1.03	(0.70, 1.51)	0.92	(0.67, 1.22)	0.89	(0.55, 1.34)	0.63	(0.16, 1.78)	0.92	(0.67, 1.23)	1.25	(0.94, 1.63)	0.91	(0.58, 1.33)	0.65	(0.19, 1.58)
Unemployed population (%)	**1.21**	**(1.02, 1.42)**	1.01	(0.89, 1.14)	1.00	(0.84, 1.19)	0.86	(0.53, 1.35)	**1.19**	**(1.04, 1.35)**	1.03	(0.92, 1.16)	0.93	(0.79, 1.08)	0.70	(0.44, 1.06)
Overcrowded households (%)^b^	0.93	(0.77, 1.10)	1.10	(0.95, 1.26)	0.95	(0.79, 1.15)	1.06	(0.66, 1.62)	0.92	(0.79, 1.05)	1.11	(0.97, 1.26)	**1.28**	**(1.07, 1.51)**	1.31	(0.80, 2.00)
Social fragmentation score																
Population mobility (%)^b^	**0.81**	**(0.67, 0.96)**	0.97	(0.84, 1.12)	1.11	(0.92, 1.33)	0.91	(0.55, 1.43)	0.97	(0.84, 1.12)	1.03	(0.91, 1.17)	1.13	(0.94, 1.34)	1.05	(0.64, 1.64)
Single-person households (%)^b^	1.14	(0.98, 1.33)	0.96	(0.84, 1.09)	0.92	(0.77, 1.11)	0.95	(0.56, 1.56)	1.11	(0.98, 1.25)	1.06	(0.94, 1.19)	1.00	(0.83, 1.19)	0.90	(0.53, 1.42)
Households privately renting (%)^b^	1.15	(0.92, 1.40)	1.07	(0.90, 1.26)	**1.48**	**(1.18, 1.84)**	1.83	(0.99, 3.19)	**1.28**	**(1.08, 1.52)**	1.03	(0.88, 1.21)	1.13	(0.94, 1.37)	1.33	(0.74, 2.25)
Unmarried adults (%)^b^	1.06	(0.86, 1.28)	1.03	(0.87, 1.21)	1.08	(0.85, 1.36)	1.50	(0.81, 2.59)	0.93	(0.79, 1.09)	0.92	(0.80, 1.07)	0.99	(0.80, 1.21)	1.15	(0.62, 1.96)
Social class IV and V households (%)^c^	0.79	(0.61, 1.00)	1.09	(0.89, 1.31)	1.30	(0.98, 1.69)	1.02	(0.46, 2.02)	0.90	(0.73, 1.08)	1.16	(0.96, 1.38)	1.08	(0.81, 1.36)	0.92	(0.42, 1.73)
Social housing (%)	1.02	(0.68, 1.41)	1.14	(0.84, 1.52)	**1.67**	**(1.06, 2.55)**	2.61	(0.64, 7.49)	1.16	(0.84, 1.55)	0.90	(0.66, 1.21)	1.35	(0.91, 2.01)	2.24	(0.63, 6.20)
Population with limiting long-term illness (%)	**1.25**	**(1.06, 1.46)**	1.08	(0.95, 1.22)	1.11	(0.94, 1.31)	1.55	(0.97, 2.34)	**1.18**	**(1.04, 1.33)**	1.11	(0.99, 1.24)	1.05	(0.89, 1.23)	1.21	(0.77, 1.82)
Lone-parent households (%)	**1.23**	**(1.04, 1.47)**	1.15	(0.99, 1.31)	0.83	(0.69, 1.00)	1.08	(0.65, 1.70)	**1.18**	**(1.03, 1.35)**	**1.18**	**(1.04, 1.34)**	1.15	(0.96, 1.35)	1.04	(0.62, 1.64)
Population other than White British (%)	**0.86**	**(0.75, 0.99)**	**0.82**	**(0.74, 0.91)**	**0.83**	**(0.72, 0.96)**	1.09	(0.75, 1.52)	**0.84**	**(0.75, 0.94)**	**0.85**	**(0.77, 0.94)**	**0.82**	**(0.73, 0.93)**	0.95	(0.65, 1.34)
Population density (per hectare)	1.05	(0.96, 1.14)	1.01	(0.94, 1.08)	0.95	(0.86, 1.04)	0.91	(0.72, 1.15)	1.01	(0.94, 1.07)	1.02	(0.96, 1.09)	0.97	(0.89, 1.05)	0.87	(0.68, 1.09)
Travel distance to the nearest emergency department by driving	**0.87**	**(0.78, 0.97)**	**0.86**	**(0.79, 0.94)**	0.90	(0.82, 1.00)	0.87	(0.68, 1.09)	0.94	(0.87, 1.03)	**0.88**	**(0.88, 0.95)**	0.95	(0.87, 1.03)	1.00	(0.79, 1.23)

a The 95% credible intervals of rate ratios that do not include one are highlighted in bold.

b These variables were firstly log-transformed because of their skewed distributions.

c Based on the occupational status of household reference person; IV: semi-skilled and unskilled manual occupations; V: on state benefit, unemployed and lowest grade occupations.

## Discussion

Our data showed distinct spatial patterning and correlates of self-harm amongst residents of the City of Manchester municipality. Males and females aged below 65 years showed similar geographical patterning of self-harm, in contrast to no clear spatial patterning in those aged 65 years and older. In a comprehensively adjusted model, rates of self-harm were positively correlated with unemployed population, households privately renting, population with limiting long-term illness and lone-parent households, and negatively associated with an ethnicity other than White British and travel distance to the nearest emergency department. Area-level characteristics explained a large proportion (four-fifths) of the variations in area self-harm rates. Some associations appeared specific to certain age groups; for example, unemployed population was mainly associated with self-harm rates of young males and females aged 10–24 years.

### Strengths and limitations

This study was amongst the first to investigate small-area spatial patterning of self-harm hospital presentations in an urban setting, based on a comprehensive dataset that included all emergency department attendances for self-harm. It also examined a wider range of potential spatial correlates of self-harm than most previous studies. The study, however, did have several limitations. First, it was restricted to hospital presenting self-harm episodes and the factors influencing the likelihood of emergency department attendance do not depend only on the severity of injuries. Second, self-harm presentations to emergency departments beyond the City of Manchester municipal boundary may have led to the underestimation of the municipality's self-harm rate. This underestimation may have been more marked towards the periphery of the municipality's boundary, although the maps showed no indication of lower incidence rates in such localities. Third, individuals with no fixed abode were excluded from the analysis and this may contribute to an underestimation of self-harm rates. However, the number of individuals with no fixed abode (*n* = 366) was small compared to our overall sample (*n* = 14 771) and thus the impact is assumed to be limited. Fourth, people aged 10–44 years accounted for most (81.6%) self-harm presentations; comparatively, the number of self-harm presentations was smaller in the older populations, which would result in lower statistical power to detect associations in these groups. Fifth, area-based socioeconomic characteristics extracted from the 2001 census data would not capture any variations across the study period. In addition, we did not include some area characteristics such as the prevalence of mental disorders and substance use disorders for which data were not available, although the population with long-term illness would capture some of this. Finally, as this was an ecological study, its findings may not apply at the individual level, and furthermore, causal associations cannot be inferred.

### Comparison with previous findings

Several previous studies from Edinburgh, UK (Buglass and Duffy, 1978), London, Canada (Jarvis *et al*., 1982) and London, England (Polling *et al*., 2019) focusing on area-level self-harm rates in relation to distance from a city centre showed inconsistent results. Our data showed no clear pattern of self-harm rates in the inner city districts around the city centre. In addition, we found no association of self-harm rates with population density, an indicator of an area's urbanisation level.

The spatial patterning of self-harm across the City of Manchester municipality's population may be attributable to several factors. Higher self-harm rate areas were characterised by high deprivation level, in keeping with the findings from an earlier study from Manchester (Johnston *et al*., 2006) and other studies from London (Congdon, 1996; Polling *et al*., 2019), Bristol (Gunnell *et al*., 1995) and Edinburgh (Buglass and Duffy, 1978), UK. In the City of Manchester municipality, social housing was mainly located in the north, east and far southern zones of the municipality (Manchester City Council, 2007), approximately corresponding to areas with above average self-harm rates. Thus, some of the spatial concentration of high self-harm rates may reflect the concentration of risk associated with the deprived population living in social housing. This is in keeping with our findings of an association between social housing and self-harm rate in the unadjusted model, which was attenuated after adjusting for other ecological variables, including deprivation. A previous study of suicide from Hong Kong showed similar findings (Hsu *et al*., 2015).

Our data showed no association of self-harm rates with social fragmentation score. The four component variables of the social fragmentation score showed different associations with self-harm rates, which were attenuated or changed direction after adjusting for other variables. Furthermore, the model with the component variables but not the social fragmentation score fitted the data better than the model with social fragmentation score but not its component variables. These findings may cast some doubt as to whether or not each component variable reflects the same concept of social fragmentation in relation to the risk of self-harm in the study city. Alternatively, the inconsistent associations between individual social fragmentation indicators and self-harm may be due to the specific context of the City of Manchester municipality, where ethnicity may confound some of the associations. Our data showed that ethnic minority, indicated by the percentage of the population other than White British, was positively correlated with population mobility and households privately renting, and negatively correlated to self-harm rates; therefore, these may attenuate the associations between self-harm and the two social fragmentation indicators.

The percentage of the non-White-British population was negatively associated with self-harm rate, in contrast to no association found in previous area-level analyses from Manchester (Johnston *et al*., 2006) and London (Polling *et al*., 2019), UK. The difference may be attributable to a marked increase in the ethnic minority population in Manchester over the study period, when the percentage of the non-White-British population increased by around 60% in 2001–2011 (ESRC Centre on Dynamics of Ethnicity (CoDE), 2013). In 2001, the largest ethnic minority groups were Pakistani (5.9%), Black Caribbean (2.3%) and Black African (1.7%) in the City of Manchester, whilst in 2011, they were Pakistani (8.5%), Black African (5.1%) and Chinese (2.7%). One previous study indicated ethnic minority as a risk factor for self-harm in neighbourhoods with low minority populations whilst a protective factor in neighbourhoods with large minority populations (Neeleman *et al*., 2001). An alternative possibility is that ethnic minority groups may be less likely to seek treatments than White counterparts (Cooper *et al*., 2010), and this may contribute to an association between lower area self-harm rates and a higher percentage of non-White-British population. Future studies into ethnicity and self-harm should consider variations in risk at both individual and area levels as well as ethnic variations in help-seeking.

In accordance with a recent study (O'Farrell *et al*., 2015), we found lower self-harm rates in areas with longer travel distance to emergency departments. The result may imply lower case ascertainment in these areas, where people who self-harmed may attend an emergency department outside the study region or, alternatively, did not seek treatment at the emergency departments due to the barrier of longer travel distance.

We found similar spatial patterning of self-harm across sex/age groups except the elderly group, which showed no obvious spatial patterning. A few previous studies that investigated sex- and age-specific spatial patterning of suicide similarly showed a higher degree of spatial variations in younger than older groups (Chang *et al*., 2011; Lin *et al*., 2019). Our regression analyses suggested that younger people living in areas characterised by high levels of unemployment, limiting longstanding illness, lone-parent households and White-British ethnicity had higher rates of self-harm. Future research into self-harm across sex/age groups considering area-level contextual factors is needed.

## Conclusions

Our data showed above average rates of self-harm in areas characterised as predominantly socioeconomically deprived, socially fragmented and ethnically White British in the City of Manchester municipality. The city centre and relatively affluent or ethnically mixed areas showed below average rates. Although the City of Manchester municipality is one of the most deprived local authority areas in England, small areas' socioeconomic characteristics still account for a large proportion of variability in self-harm incidence. These findings have implications for targeting prevention and intervention resources at high rate areas to reduce self-harm and addressing social and material issues, particularly amongst young people. Targets for area-based interventions might include tackling the causes and consequences of joblessness, better treatment of long-term illness and consideration of the accessibility to health and social services. Future research into self-harm considering both individual- and area-level factors is also needed.

## Supplementary material

For supplementary material accompanying this paper visit https://doi.org/10.1017/S2045796019000696. click here to view supplementary material

## Data

The data were from The Manchester Self-Harm Project. We were not permitted to provide the data.
