# Spatial patterning and correlates of self-harm in Manchester, England – CORRIGENDUM

**DOI:** 10.1017/S2045796020000219

**Published:** 2020-02-19

**Authors:** Chien-Yu Lin, Harriet Bickley, Caroline Clements, Roger T. Webb, David Gunnell, Chia-Yueh Hsu, Shu-Sen Chang, Nav Kapur

**Affiliations:** 1 Institute of Health Behaviors and Community Sciences, College of Public Health, National Taiwan University, Taipei, Taiwan; 2 Graduate School of Sport Sciences, Waseda University, Tokorozawa, Japan; 3 Division of Psychology & Mental Health, Centre for Mental Health and Safety, The University of Manchester, Manchester, UK; 4 Manchester Academic Health Sciences Centre (MAHSC), Manchester, UK; 5 National Institute for Health Research Greater Manchester Patient Safety Translational Research Centre, Manchester, UK; 6 Bristol Medical School, Population Health Sciences, University of Bristol, Bristol, UK; 7 National Institute for Health Research Biomedical Research Centre at the University Hospitals Bristol NHS Foundation Trust and the University of Bristol, Bristol, UK; 8 Department of Psychiatry, Wan Fang Hospital, Taipei Medical University, Taipei, Taiwan; 9 Greater Manchester Mental Health NHS Foundation Trust, Manchester, UK; 10 Department of Psychiatry, School of Medicine, College of Medicine, Taipei Medical University, Taipei, Taiwan; 11 Psychiatric Research Center, Wan Fang Hospital, Taipei Medical University, Taipei, Taiwan

The authors would like to correct an error in affiliation 10. The correct affiliation is “Department of Psychiatry, School of Medicine, College of Medicine, Taipei Medical University, Taipei, Taiwan”. The authors apologise for this error.
