# Supplementary material for: Spatial patterning and correlates of self-harm in Manchester, England
Source: Epidemiol Psychiatr Sci. 2019 Nov 19;29:e72. doi: 10.1017/S2045796019000696 (PMC8061130; doi:10.1017/S2045796019000696)
Supplement: Supplementary file 1 [file S2045796019000696sup001.docx]

# Appendix Figure 1. The map of posterior probability of smoothed standardised incidence ratios of self-harm greater than one (i.e. the probability of above-average self-harm rates, ranging from 0 to 1) across 258 Lower Super Output Areas in the City of Manchester municipality.


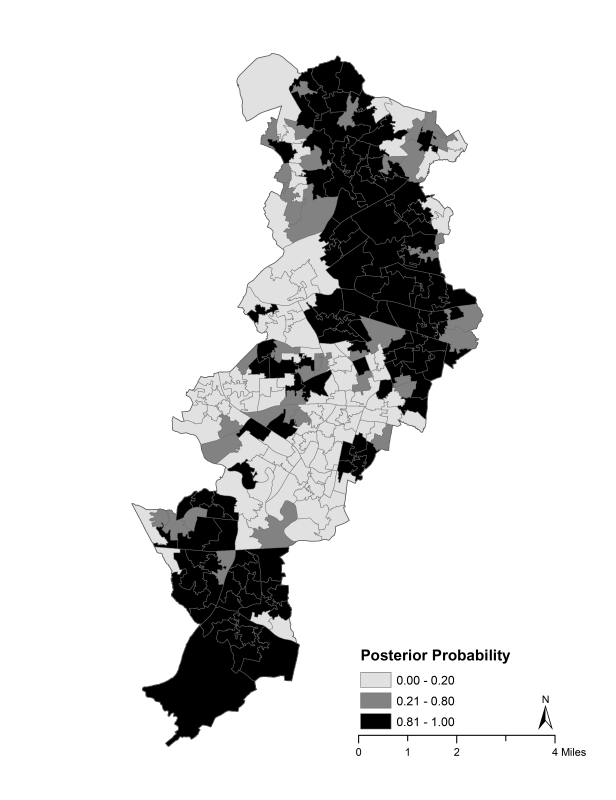


# Appendix Figure 2. Maps of smoothed standardised incidence ratios (SIRs) for index self-harm episode (in people aged 10 or more years) for specific suicide method across 258 Lower Super Output Areas in the City of Manchester municipality, 2003-2013.


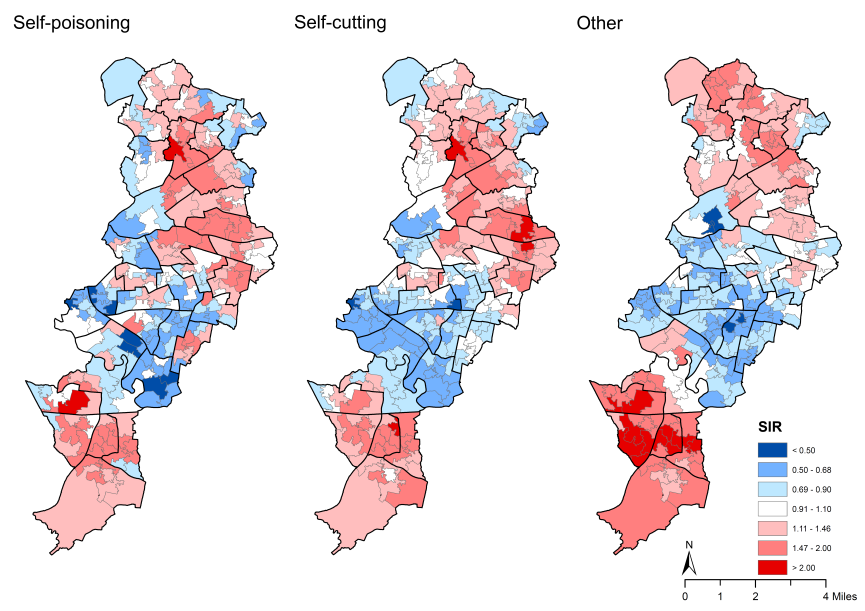


Note: The boundaries of census area statistics ward (casward; n=33) were highlighted in bold black.

# Appendix Figure 3. Maps of the Townsend deprivation index (a-d), the social fragmentation score (e-h), and other area-level characteristics (i-o) across 258 Lower Super Output Areas in the City of Manchester municipality.


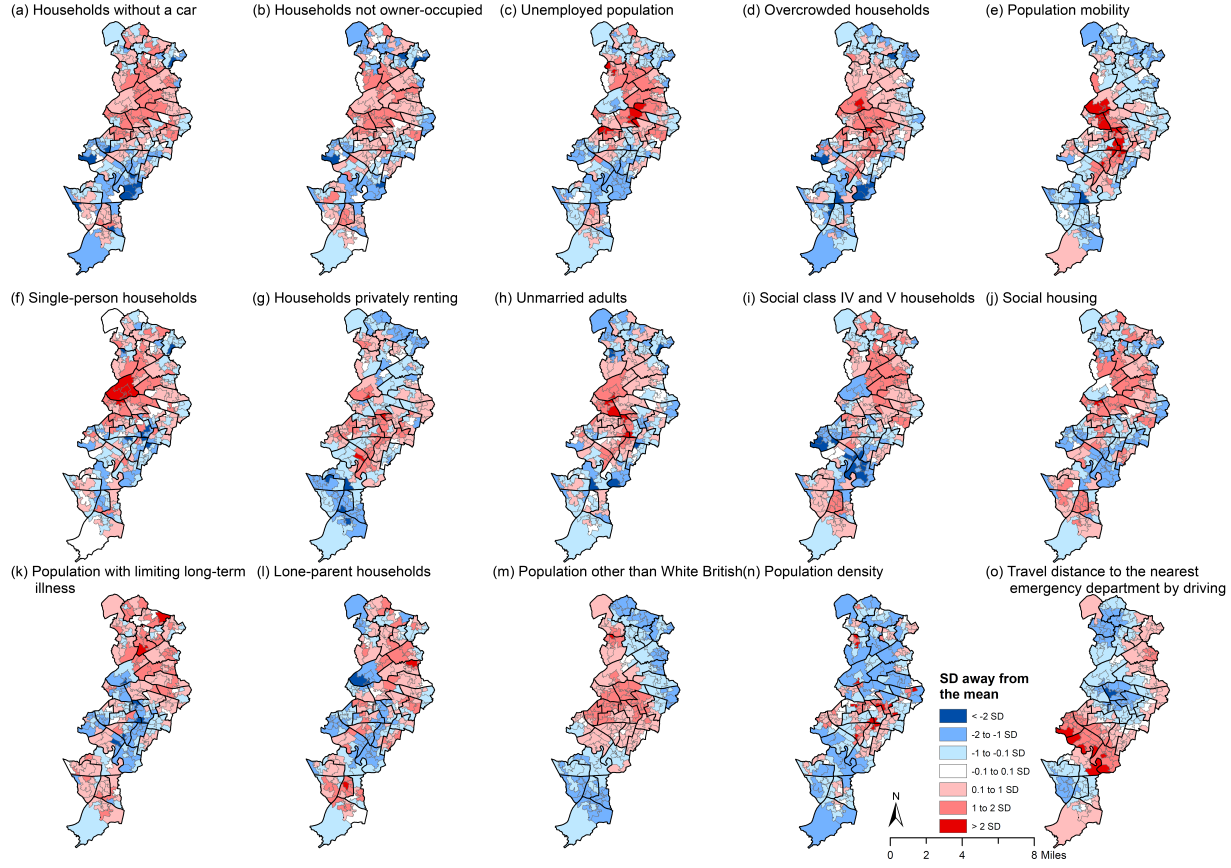


Note: The boundaries of census area statistics ward (casward; n=33) were highlighted in bold black.

# Appendix Table 1. Spearman’s correlation between area socioeconomic characteristics and self-harm.

|  | a | b | c | d | e | f | g | h | i | j | k | l | m | n | o | Self-harm |
| --- | --- | --- | --- | --- | --- | --- | --- | --- | --- | --- | --- | --- | --- | --- | --- | --- |
| Townsend deprivation index |  |  |  |  |  |  |  |  |  |  |  |  |  |  |  | 0.47** |
| a. Households without a car (%) | 1 |  |  |  |  |  |  |  |  |  |  |  |  |  |  | 0.62** |
| b. Households not owner-occupied (%) | 0.86** | 1 |  |  |  |  |  |  |  |  |  |  |  |  |  | 0.50** |
| c. Unemployed population (%) | 0.82** | 0.73** | 1 |  |  |  |  |  |  |  |  |  |  |  |  | 0.63** |
| d. Overcrowded households (%) | 0.53** | 0.66** | 0.41** | 1 |  |  |  |  |  |  |  |  |  |  |  | 0.05 |
| Social fragmentation score |  |  |  |  |  |  |  |  |  |  |  |  |  |  |  | -0.08 |
| e. Population mobility (%) | 0.14* | 0.29** | 0.03 | 0.61** | 1 |  |  |  |  |  |  |  |  |  |  | -0.31** |
| f. Single-person households (%) | 0.56** | 0.48** | 0.31** | 0.56** | 0.26** | 1 |  |  |  |  |  |  |  |  |  | 0.29** |
| g. Households privately renting (%) | -0.16* | -0.12* | -0.19** | 0.46** | 0.71** | 0.08 | 1 |  |  |  |  |  |  |  |  | -0.48** |
| h. Unmarried adults (%) | 0.49** | 0.68** | 0.26** | 0.71** | 0.66** | 0.58** | 0.33** | 1 |  |  |  |  |  |  |  | 0.08 |
| i. Social class IV and V households (%)^a^ | 0.81** | 0.69** | 0.80** | 0.18** | -0.27** | 0.22** | -0.49** | 0.11 | 1 |  |  |  |  |  |  | 0.76** |
| j. Social housing (%) | 0.80** | 0.82** | 0.77** | 0.29** | -0.13** | 0.39** | -0.56** | 0.33** | 0.85** | 1 |  |  |  |  |  | 0.71** |
| k. Population with limiting long-term illness (%) | 0.56** | 0.38** | 0.55** | -0.04 | -0.50** | 0.36** | -0.57** | -0.06 | 0.76** | 0.67** | 1 |  |  |  |  | 0.76** |
| l. Lone-parent households (%) | 0.55** | 0.47** | 0.68** | -0.12* | -0.39** | -0.08 | -0.56** | -0.05 | 0.82** | 0.74** | 0.63** | 1 |  |  |  | 0.73** |
| m. Population other than White British (%) | 0.07 | 0.16** | 0.22** | 0.49** | 0.57** | 0.04 | 0.58** | 0.26** | -0.19** | -0.10 | -0.43** | -0.22** | 1 |  |  | -0.37** |
| n. Population density (per hectare) | -0.07 | -0.03 | -0.07 | 0.17 | 0.25** | -0.24** | 0.38** | 0.07 | -0.15* | -0.23** | -0.40** | -0.16** | 0.27** | 1 |  | -0.27** |
| o. Travel distance to the nearest emergency department by driving | -0.45** | -0.33** | -0.42** | -0.25** | -0.05 | -0.15* | 0.09 | -0.05 | -0.35** | -0.32** | -0.17** | -0.21** | -0.16* | -0.02 | 1 | -0.31** |

^a^ Based on the occupational status of household reference person; IV: semi-skilled and unskilled manual occupations; V: on state benefit, unemployed and lowest grade occupations.

# Appendix Table 2. Adjusted rate ratios (RR) and their 95% Credible Intervals (CrI)^a^ of index self-harm episode incidence associated with one standard deviation increase in levels of each of the area socioeconomic characteristics across 258 Lower Super Output Areas (LSOAs) in the City of Manchester municipality, 2003–2013. VIF: variance inflation factor.

|  | Adjusted for 15 variables | | | VIF |  | Adjusted for 14 variables | | | VIF |  | Adjusted for 13 variables | | | VIF |  | Adjusted for 12 variables | | | VIF |
| --- | --- | --- | --- | --- | --- | --- | --- | --- | --- | --- | --- | --- | --- | --- | --- | --- | --- | --- | --- |
|  | RR | (95% CrI) | |  |  | RR | (95% CrI) | |  |  | RR | (95% CrI) | |  |  | RR | (95% CrI) | |  |
| **Townsend deprivation index** |  |  |  |  |  |  |  |  |  |  |  |  |  |  |  |  |  |  |  |
| Households without a car (%) | 1.01 | (0.88 , | 1.15) | 19.2 |  | 1.00 | (0.90 , | 1.12) | 10.8 |  | 0.99 | (0.89 , | 1.10) | 10.7 |  | 1.00 | (0.91 , | 1.10) | 9.4 |
| Households not owner-occupied (%) | 0.93 | (0.78 , | 1.13) | 30.2 |  | 0.95 | (0.79 , | 1.15) | 30.1 |  | 1.05 | (0.93 , | 1.17) | 14.7 |  | - | - | - |  |
| Unemployed population (%) | **1.10** | **(1.02 ,** | **1.19)** | 7.0 |  | **1.09** | **(1.02 ,** | **1.17)** | 7.0 |  | **1.10** | **(1.02 ,** | **1.18)** | 6.9 |  | **1.11** | **(1.03 ,** | **1.18)** | 6.5 |
| Overcrowded households (%)^b^ | 1.03 | (0.96 , | 1.12) | 6.8 |  | 1.03 | (0.95 , | 1.11) | 6.6 |  | 1.02 | (0.95 , | 1.10) | 6.5 |  | 1.04 | (0.97 , | 1.11) | 4.7 |
| **Social fragmentation score** |  |  |  |  |  |  |  |  |  |  |  |  |  |  |  |  |  |  |  |
| Population mobility (%)^b^ | 0.99 | (0.91 , | 1.07) | 8.3 |  | 0.98 | (0.91 , | 1.07) | 8.3 |  | 0.99 | (0.91 , | 1.06) | 8.3 |  | 0.99 | (0.91 , | 1.07) | 8.2 |
| Single-person households (%)^b^ | 1.06 | (0.99 , | 1.14) | 5.9 |  | 1.07 | (1.00 , | 1.14) | 4.7 |  | **1.08** | **(1.02 ,** | **1.15)** | 4.3 |  | **1.08** | **(1.01 ,** | **1.14)** | 4.0 |
| Households privately renting (%)^b^ | **1.20** | **(1.08 ,** | **1.32)** | 10.2 |  | **1.19** | **(1.08 ,** | **1.31)** | 10.1 |  | **1.12** | **(1.05 ,** | **1.19)** | 4.4 |  | **1.11** | **(1.05 ,** | **1.17)** | 3.2 |
| Unmarried adults (%)^b^ | 0.95 | (0.86 , | 1.05) | 9.3 |  | 0.95 | (0.87 , | 1.04) | 9.1 |  | 0.95 | (0.87 , | 1.04) | 9.1 |  | 0.97 | (0.89 , | 1.05) | 7.0 |
| Social class IV and V households (%)^c^ | 0.98 | (0.88 , | 1.10) | 34.5 |  | - | - | - |  |  | - | - | - |  |  | - | - | - |  |
| Social housing (%) | 1.18 | (0.99 , | 1.42) | 1.7 |  | 1.16 | (0.96 , | 1.38) | 34.0 |  | - | - | - |  |  | - | - | - |  |
| Population with limiting long-term illness (%) | **1.17** | **(1.09 ,** | **1.25)** | 6.2 |  | **1.16** | **(1.09 ,** | **1.24)** | 5.3 |  | **1.18** | **(1.11 ,** | **1.25)** | 5.2 |  | **1.18** | **(1.11 ,** | **1.26)** | 5.2 |
| Lone-parent households (%) | **1.17** | **(1.09 ,** | **1.26)** | 7.5 |  | **1.17** | **(1.08 ,** | **1.26)** | 7.2 |  | **1.20** | **(1.12 ,** | **1.28)** | 6.0 |  | **1.21** | **(1.13,** | **1.29)** | 5.8 |
| Population other than White British (%) | **0.84** | **(0.79 ,** | **0.90)** | 3.6 |  | **0.85** | **(0.79 ,** | **0.90)** | 3.6 |  | **0.85** | **(0.80 ,** | **0.91)** | 3.3 |  | **0.86** | **(0.81 ,** | **0.91)** | 3.3 |
| Population density (per hectare) | 1.00 | (0.96 , | 1.04) | 15.4 |  | 1.00 | (0.96 , | 1.04) | 1.7 |  | 1.00 | (0.96 , | 1.04) | 1.7 |  | 1.00 | (0.96 , | 1.04) | 1.7 |
| Travel distance to the nearest emergency department by driving | **0.91** | **(0.86 ,** | **0.95)** | 1.7 |  | **0.90** | **(0.86 ,** | **0.95)** | 1.7 |  | **0.91** | **(0.86 ,** | **0.95)** | 1.7 |  | **0.91** | **(0.86 ,** | **0.95)** | 1.6 |

^a^ 95% credible intervals of rate ratios that do not include one are highlighted in bold.

^b^ These variables were firstly log-transformed because of their skewed distributions.

^c^ Based on the occupational status of household reference person; IV: semi-skilled and unskilled manual occupations; V: on state benefit, unemployed and lowest grade occupations.

# Appendix Table 3. Summary statistics of the distribution of smoothed standardised incidence ratios for index self-harm episode in people aged 10 years or above across 258 Lower Super Output Areas in the City of Manchester municipality, 2003-2013

|  | Mean | SD | 5% | Median | 95% | Mid-90% ratio^a^ | Moran’s I | p value |
| --- | --- | --- | --- | --- | --- | --- | --- | --- |
| All sex/age groups combined | 1.06 | 0.43 | 0.45 | 1.01 | 1.84 | 4.10 | 0.51 | <0.001 |
| By sex/age group |  |  |  |  |  |  |  |  |
| Males |  |  |  |  |  |  |  |  |
| All ages combined | 1.06 | 0.50 | 0.38 | 0.97 | 1.99 | 5.30 | 0.48 | <0.001 |
| Aged 10-24 | 1.28 | 0.67 | 0.41 | 1.18 | 2.46 | 6.05 | 0.28 | <0.001 |
| Aged 25-44 | 1.05 | 0.53 | 0.31 | 0.97 | 2.08 | 6.61 | 0.43 | <0.001 |
| Aged 45-64 | 0.99 | 0.30 | 0.59 | 0.95 | 1.60 | 2.72 | 0.14 | <0.001 |
| Aged 65+ | 1.00 | 0.07 | 0.92 | 0.99 | 1.16 | 1.26 | 0.08 | 0.023 |
| Females |  |  |  |  |  |  |  |  |
| All ages combined | 1.06 | 0.38 | 0.53 | 1.37 | 2.03 | 3.81 | 0.40 | <0.001 |
| Aged 10-24 | 1.21 | 0.47 | 0.54 | 1.17 | 2.09 | 3.87 | 0.30 | <0.001 |
| Aged 25-44 | 1.02 | 0.37 | 0.45 | 1.01 | 1.71 | 3.78 | 0.29 | <0.001 |
| Aged 45-64 | 1.01 | 0.24 | 0.67 | 0.97 | 1.48 | 2.21 | 0.20 | <0.001 |
| Aged 65+ | 1.00 | 0.08 | 0.92 | 0.97 | 1.13 | 1.23 | -0.01 | 0.423 |
| By self-harm method |  |  |  |  |  |  |  |  |
| Self-poisoning | 1.06 | 0.43 | 0.46 | 1.01 | 1.81 | 3.95 | 0.50 | <0.001 |
| Self-cutting | 1.05 | 0.36 | 0.57 | 1.01 | 1.70 | 2.98 | 0.32 | <0.001 |
| Other | 1.04 | 0.44 | 0.55 | 0.93 | 1.97 | 3.57 | 0.31 | <0.001 |

^a^ Differences over the 90% mid-range, i.e. the values at 95% divided by the values at 5%.

# Appendix Table 4. Rate ratios (RR) and 95% Credible Intervals (CrI)^a^ of index self-harm incidence (in people aged 10 or more years) associated with one standard deviation increase in levels of each of the area socioeconomic characteristics across 258 Lower Super Output Areas in the City of Manchester municipality, 2003-2013: sensitivity analysis using the Index of Multiple Deprivation 2010

|  | Unadjusted | | |  | Adjusted for 9 variables | | |  | Adjusted for 18 variables | | |
| --- | --- | --- | --- | --- | --- | --- | --- | --- | --- | --- | --- |
|  | RR | 95% CrI | |  | RR | 95% CrI | |  | RR | 95% CrI | |
| **The Index of Multiple Deprivation** | **1.45** | **(1.39 ,** | **1.50)** |  | **1.26** | **(1.17 ,** | **1.03)** |  |  |  |  |
| Income Deprivation Domain | **1.36** | **(1.31 ,** | **1.42)** |  |  |  |  |  | 0.98 | (0.88 , | 1.10) |
| Employment Deprivation Domain | **1.42** | **(1.36 ,** | **1.47)** |  |  |  |  |  | **1.13** | **(1.01 ,** | **1.27)** |
| Health Deprivation and Disability Domain | **1.38** | **(1.33 ,** | **1.43)** |  |  |  |  |  | **1.15** | **(1.08 ,** | **1.22)** |
| Education, Skills and Training Deprivation Domain | **1.43** | **(1.36 ,** | **1.50)** |  |  |  |  |  | 0.99 | (0.91 , | 1.07) |
| Barriers to Housing and Services Domain | 0.98 | (0.93 , | 1.04) |  |  |  |  |  | 1.00 | (0.96 , | 1.03) |
| Crime Domain | **1.10** | **(1.05 ,** | **1.15)** |  |  |  |  |  | 1.00 | (0.97 , | 1.03) |
| Living Environment Deprivation Domain | **0.94** | **(0.89 ,** | **0.99)** |  |  |  |  |  | 0.99 | (0.94 , | 1.03) |
| **Social fragmentation score** | 1.01 | (0.99 , | 1.03) |  | **1.02** | **(1.01 ,** | **1.03)** |  |  |  |  |
| Population mobility (%)* | **0.91** | **(0.85 ,** | **0.98)** |  |  |  |  |  | 0.98 | (0.92 , | 1.06) |
| Single-person households (%)* | **1.16** | **(1.10 ,** | **1.21)** |  |  |  |  |  | **1.11** | **(1.04 ,** | **1.18)** |
| Households living in private rented households (%)* | **0.86** | **(0.81 ,** | **0.93)** |  |  |  |  |  | **1.16** | **(1.07 ,** | **1.25)** |
| Unmarried adults (%)* | **1.17** | **(1.10 ,** | **1.24)** |  |  |  |  |  | 0.93 | (0.86 , | 1.00) |
| Social class IV and V households (%) | **1.38** | **(1.31 ,** | **1.45)** |  | **0.90** | **(0.82 ,** | **0.98)** |  | 0.99 | (0.90 , | 1.09) |
| Social housing (%) | **1.34** | **(1.29 ,** | **1.40)** |  | 0.99 | (0.93 , | 1.06) |  | 1.03 | (0.92 , | 1.15) |
| Population with limiting long-term illness (%) | **1.38** | **(1.32 ,** | **1.45)** |  | **1.19** | **(1.12 ,** | **1.27)** |  | 1.06 | (0.98 , | 1.13) |
| Lone-parent households (%) | **1.31** | **(1.25 ,** | **1.37)** |  | **1.15** | **(1.07 ,** | **1.22)** |  | **1.20** | **(1.12 ,** | **1.29)** |
| Population other than White-British (%) | **0.87** | **(0.79 ,** | **0.95)** |  | **0.93** | **(0.89 ,** | **0.97)** |  | **0.90** | **(0.83 ,** | **0.96)** |
| Population density (per hectare) | **0.95** | **(0.90 ,** | **1.00)** |  | 1.01 | (0.97 , | 1.04) |  | 0.99 | (0.96 , | 1.03) |
| Travel distance to the nearest emergency department by driving | 0.95 | (0.86 , | 1.06) |  | **0.90** | **(0.86 ,** | **0.95)** |  | **0.92** | **(0.88 ,** | **0.97)** |

# Appendix Table 5. Adjusted rate ratios (RR) and 95% Credible Intervals (CrI)^a^ of index self-harm incidence (in people aged 10 or more years) associated with quartiles of increasing levels and one standard deviation (SD) increase in each of the area socioeconomic characteristics across 258 Lower Super Output Areas in the City of Manchester municipality, 2003-2013. DIC: deviance information criterion.

|  | Models including the variable as a categorical variable^b^ | | | | | | | | | | | | | | |  | Model including all variables as continuous variables | | | | |
| --- | --- | --- | --- | --- | --- | --- | --- | --- | --- | --- | --- | --- | --- | --- | --- | --- | --- | --- | --- | --- | --- |
|  | Quartile 1 |  | Quartile 2 | | |  | Quartile 3 | | |  | Quartile 4 | | |  | DIC |  | 1 SD increase | | |  | DIC |
|  | RR |  | RR | 95% CrI | |  | RR | 95% CrI | |  | RR | 95% CrI | |  |  |  | RR | 95% CrI | |  |  |
| **Townsend deprivation index** |  |  |  |  |  |  |  |  |  |  |  |  |  |  |  |  |  |  |  |  |  |
| Households without a car (%) | 1.00 |  | 1.02 | (0.89 , | 1.14) |  | 0.97 | (0.83 , | 1.13) |  | 0.89 | (0.71 , | 1.09) |  | 1907.3 |  | 1.01 | (0.88 , | 1.15) |  | 1908.1 |
| Households not owner-occupied (%) | 1.00 |  | 1.03 | (0.90 , | 1.17) |  | 0.94 | (0.79 , | 1.12) |  | 0.91 | (0.72 , | 1.13) |  | 1908.1 |  | 0.93 | (0.78 , | 1.13) |  | 1908.1 |
| Unemployed population (%) | 1.00 |  | **1.27** | **(1.14 ,** | **1.41)** |  | **1.37** | **(1.19 ,** | **1.55)** |  | **1.40** | **(1.17 ,** | **1.65)** |  | **1901.8** |  | **1.10** | **(1.02 ,** | **1.19)** |  | 1908.1 |
| Overcrowded households (%)^c^ | 1.00 |  | 1.05 | (0.95 , | 1.15) |  | **1.10** | **(0.98 ,** | **1.24)** |  | 1.09 | (0.93 , | 1.27) |  | 1908.8 |  | 1.03 | (0.96 , | 1.12) |  | 1908.1 |
| **Social fragmentation score** |  |  |  |  |  |  |  |  |  |  |  |  |  |  |  |  |  |  |  |  |  |
| Population mobility (%)^c^ | 1.00 |  | 1.03 | (0.94 , | 1.12) |  | 1.06 | (0.95 , | 1.17) |  | 1.12 | (0.96 , | 1.29) |  | 1909.0 |  | 0.99 | (0.91 , | 1.07) |  | 1908.1 |
| Single-person households (%)^c^ | 1.00 |  | **1.12** | **(1.01 ,** | **1.22)** |  | 1.13 | (1.01 , | 1.26) |  | 1.10 | (0.94 , | 1.29) |  | 1907.8 |  | 1.06 | (0.99 , | 1.14) |  | 1908.1 |
| Households privately renting (%)^c^ | 1.00 |  | 0.99 | (0.90 , | 1.10) |  | **1.21** | **(1.04 ,** | **1.39)** |  | 1.22 | (0.97 , | 1.55) |  | 1910.6 |  | **1.20** | **(1.08 ,** | **1.32)** |  | 1908.1 |
| Unmarried adults (%)^c^ | 1.00 |  | 1.01 | (0.91 , | 1.13) |  | 1.02 | (0.90 , | 1.15) |  | 1.03 | (0.88 , | 1.21) |  | 1910.2 |  | 0.95 | (0.86 , | 1.05) |  | 1908.1 |
| Social class IV and V households (%)^d^ | 1.00 |  | 1.07 | (0.95 , | 1.21) |  | 0.96 | (0.81 , | 1.14) |  | 0.89 | (0.72 , | 1.11) |  | **1904.9** |  | 0.98 | (0.88 , | 1.10) |  | 1908.1 |
| Social housing (%) | 1.00 |  | 1.07 | (0.96 , | 1.18) |  | 1.11 | (0.92 , | 1.32) |  | 1.10 | (0.84 , | 1.39) |  | 1910.0 |  | 1.18 | (0.99 , | 1.42) |  | 1908.1 |
| Population with limiting long-term illness (%) | 1.00 |  | 1.14 | (1.03 , | 1.27) |  | **1.20** | **(1.05 ,** | **1.37)** |  | 1.23 | (1.05 , | 1.43) |  | 1914.6 |  | **1.17** | **(1.09 ,** | **1.25)** |  | 1908.1 |
| Lone-parent households (%) | 1.00 |  | **1.18** | **(1.04 ,** | **1.32)** |  | **1.24** | **(1.08 ,** | **1.42)** |  | **1.29** | **(1.08 ,** | **1.50)** |  | 1911.3 |  | **1.17** | **(1.09 ,** | **1.26)** |  | 1908.1 |
| Population other than British White (%) | 1.00 |  | 1.01 | (0.91 , | 1.11) |  | 0.90 | (0.79 , | 1.03) |  | **0.81** | **(0.68 ,** | **0.94)** |  | 1915.0 |  | **0.84** | **(0.79 ,** | **0.90)** |  | 1908.1 |
| Population density (per hectare) | 1.00 |  | 1.03 | (0.96 , | 1.12) |  | 1.06 | (0.98 , | 1.15) |  | 1.05 | (0.95 , | 1.16) |  | 1909.2 |  | 1.00 | (0.96 , | 1.04) |  | 1908.1 |
| Travel distance to the nearest emergency department by driving | 1.00 |  | 0.86 | (0.78 , | 0.94) |  | 0.85 | (0.76 , | 0.94) |  | **0.77** | **(0.67 ,** | **0.89)** |  | 1907.7 |  | **0.91** | **(0.86 ,** | **0.95)** |  | 1908.1 |

^a^ 95% credible intervals of rate ratios that do not include one are highlighted in bold.

^b^ One variable was included as a categorical variable (i.e. quartiles) at a time whilst all other variables were included and adjusted as continuous variables (i.e. z scores).

^c^ These variables were firstly log-transformed because of their skewed distributions.

^d^ Based on the occupational status of household reference person; IV: semi-skilled and unskilled manual occupations; V: on state benefit, unemployed and lowest grade occupations.
